# Icariside II Induces Apoptosis in U937 Acute Myeloid Leukemia Cells: Role of Inactivation of STAT3-Related Signaling

**DOI:** 10.1371/journal.pone.0028706

**Published:** 2012-04-06

**Authors:** Sang-Hun Kang, Soo-Jin Jeong, Sun-Hee Kim, Ji-Hyun Kim, Ji Hoon Jung, Wonil Koh, Jung Hyo Kim, Dae Keun Kim, Chang-Yan Chen, Sung-Hoon Kim

**Affiliations:** 1 College of Oriental Medicine, Kyung Hee University, Seoul, South Korea; 2 Basic Herbal Research Group, Korea Institute of Oriental Medicine, Daejeon, South Korea; 3 Chosun Nursing College, Kwangju, South Korea; 4 College of Pharmacy, Woosuk University, Wanju, South Korea; 5 Beth Israel Deaconess Medical Center, Harvard Medical School, Boston, Massachusetts, United States of America; Emory University, United States of America

## Abstract

**Background:**

The aim of this study is to determine anti-cancer effect of Icariside II purified from the root of *Epimedium koreanum* Nakai on human acute myeloid leukemia (AML) cell line U937.

**Methodology/Principal Findings:**

Icariside II blocked the growth U937 cells in a dose- and time-dependent manner. In this anti-proliferation process, this herb compound rendered the cells susceptible to apoptosis, manifested by enhanced accumulation of sub-G1 cell population and increased the terminal deoxynucleotidyl transferase dUTP nick end labeling (TUNEL)-positive cells. Icariside II was able to activate caspase-3 and cleaved poly (ADP-ribose) polymerase (PARP) in a time-dependent manner. Concurrently, the anti-apoptotic proteins, such as bcl-x_L_ and survivin in U937 cells, were downregulated by Icariside II. In addition, Icariside II could inhibit STAT3 phosphorylation and function and subsequently suppress the activation of Janus activated kinase 2 (JAK2), the upstream activators of STAT3, in a dose- and time-dependent manner. Icariside II also enhanced the expression of protein tyrosine phosphatase (PTP) SH2 domain-containing phosphatase (SHP)-1, and the addition of sodium pervanadate (a PTP inhibitor) prevented Icariside II-induced apoptosis as well as STAT3 inactivation in STAT3 positive U937 cells. Furthermore, silencing SHP-1 using its specific siRNA significantly blocked STAT3 inactivation and apoptosis induced by Icariside II in U937 cells.

**Conclusions/Significance:**

Our results demonstrated that via targeting STAT3-related signaling, Icariside II sensitizes U937 cells to apoptosis and perhaps serves as a potent chemotherapeutic agent for AML.

## Introduction

Icariside II, a flavonoid compound, is derived from the stems and leaves of *Epimedium koreanum* that has been traditionally utilized for neurasthenia, amnesia and impotence in Oriental medicine [1], [2]. The other compounds from *E. koreanum* exerted various biological activities. For instance, icariin could stimulate angiogenesis by activating the extracellular signal-related kinase (ERK) and phosphatidylinositol 3-kinase (PI3K)/AKT/endothelial nitric oxide synthase (eNOS)-dependent signal pathways in human endothelial cells [3]. Also, ikarisoside A inhibited osteoclatogenic differentiation via c-Jun N-terminal kinase (JNK) and nuclear factor kappa B (NF-κB) in RAW 264.7 cells [4]. We and others recently reported that Icariside II appeared to possess anti-cancer activity against multiple myeloma [5], prostate cancer [6] and osteosarcoma cells [7].

Acute myeloid leukemia (AML) is an aggressive malignancy characterized by the rapid growth of abnormal white blood cells (WBCs). AML is primarily treated by chemotherapy and rarely applied by radiotherapy [8]. Although various chemotherapeutic agents such as cytarabine, daunorubicin and idarubicin have been developed for AML treatment, they can affect even normal cells to cause unpleasant side effects such as anemia, bleeding and infection. In recent studies, many groups have suggested the potential of natural products as potent chemotherapeutic drugs for AML to improve the therapeutic efficacy and lower the side effects. For instance, wogonin, an active compound in *Scutellaria baicalensis*, induced apoptosis by inhibiting telomerase activity in HL-60 AML cells [9] and ajoene, a natural garlic compound, was suggested as an anti-leukemic agent for AML therapy [10]. In addition, corchorusin-D, a saikosaponin-like compound isolated from *Corchorus acutangulus*, targeted mitochondrial apoptotic pathways in HL-60 and U937 cells [11].

In the present study, the underlying anti-cancer mechanisms of Icariside II in U937 AML cells were investigated using cytotoxicity assay, cell cycle analysis, terminal deoxynucleotidyl transferase dUTP nick end labeling (TUNEL) assay, Western blotting and electrophoretic mobility shift assay (EMSA).

## Results

### Icariside II exerted anti-proliferation activity in U937 cells

**Figure 1:**
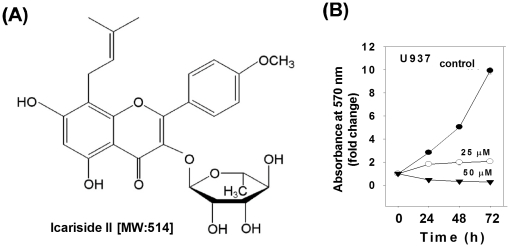
Effect of Icariside II on the proliferation of U937 cells. (A) Chemical structure of Icariside II. (B) Cells were treated with Icariside II (0, 25 or 50 µM) for 0, 24, 48 or 72 h and subjected to MTT assay to analyze cell proliferation. Data are presented as means ± SD for triplicate experiments.

Icariside II, a flavonoid compound, is purified from the stems and leaves of *Epimedium koreanum* (Fig. 1A). To evaluate the effect of this herb compound on the proliferation of U937 cells, MTT assay was performed. U937 cells were treated with Icariside II at the concentrations of 0, 25 or 50 µM for 0, 24, 48 or 72 h, respectively. The treatment with Icariside II dramatically inhibited the proliferation of U937 cells in a dose- and time-dependent manner (Fig. 1B).

### Icariside II induced apoptotic cell death in U937 cells

**Figure 2:**
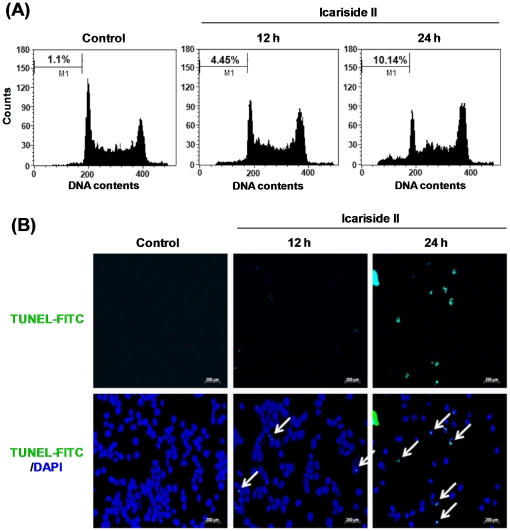
Effect of Icariside II on apoptosis induction in U937 cells. Cells were treated with Icariside II (50 µM) for 24 h. (A) The treated cells were fixed with 70% ethanol, stained with propidium iodide (PI) and analyzed the sub-G1 apoptotic cells by flow cytometry. (B) TUNEL staining was performed by using Dead End™ fluorometric TUNEL assay kit (Promega) and visualized under fluorescence microscopy (×200). Arrows indicate TUNEL (FITC)-stained cells. Representative results of three independent experiments are shown for each experiment.

To examine whether Icariside II could induce apoptosis in U937 cells, sub-G1 DNA contents were measured by DNA fragmentation analysis. Icariside II increased the accumulation of sub-G1 population to 10.14%, compared that of the control (1.1%) at 24 h after the treatment (Fig. 2A). TUNEL assay further confirmed the occurrence of apoptosis after U937 cells were treated with Icariside that increased the number of fluorescein isothiocyanate (FITC)-stained, TUNEL positive cells in a time-dependent manner (Fig. 2B).

### Icariside II regulated apoptosis-related proteins in U937 cells

**Figure 3:**
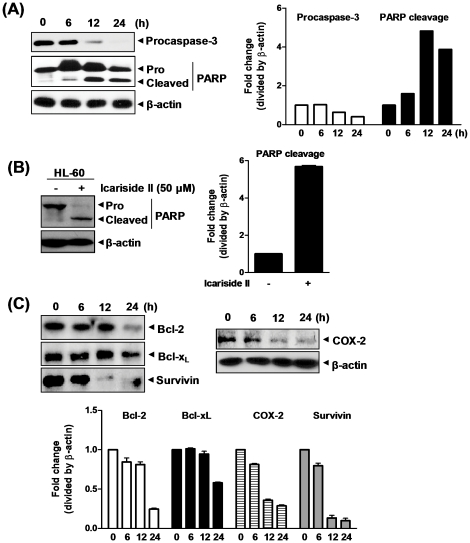
Effect of Icariside II on apoptosis-related proteins in U937 cells. (A) U937 cells were treated with Icariside II (50 µM) for 0, 6, 12 or 24 h. Cell lysates were prepared and subjected to Western blotting for procaspase-3 and PARP. (B) HL-60 cells were treated with or without Icariside II (50 µM) for 24 h. Western blot analysis was performed for PARP. (C) U937 cells were treated with Icariside II (50 µM) for 0, 6, 12 or 24 h. Western blotting was performed for bcl-2, bcl-x_L_, survivin and COX-2. Representative results of three independent experiments are shown for each experiment.

Caspase-3 is a key mediator of apoptosis [12]. Icariside II strongly blocked the expression of pro caspase-3 and induced cleavage of PARP, a substrate for caspase-3, in a time-dependent manner (Fig. 3A). Icariside II also mediated PARP cleavage in another AML cell line HL-60 (Fig. 3B), confirming the ability of Icariside II to induce apoptosis in AML cells. In addition, Icariside II treatment attenuated the expression levels of anti-apoptotic proteins including bcl-2, bcl-x_L_, survivin and COX-2 in a time-dependent manner inU937 cells (Fig. 3C).

### Icariside II suppressed STAT3 activation in U937 cells

**Figure 4:**
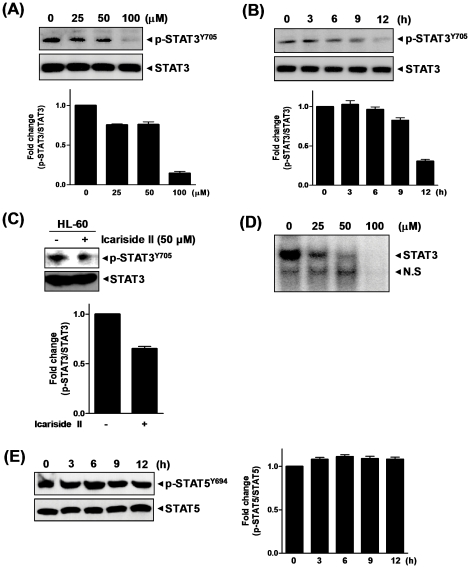
Inhibitory effect of Icariside II on activation of STAT3 in U937 cells. (A) U937 cells were treated with Icariside II (0, 25, 50 or 100 µM) for 9 h. (B) U937 cells were treated with Icariside II (50 µM) for 0, 3, 6, 9 or 12 h. Cell lysates were prepared and subjected to Western blotting for phospho-STAT3 and STAT3. (C) HL-60 cells were treated with or without Icariside II (50 µM) for 9 h. Western blotting was performed for phospho-STAT3 and STAT3. (D) U937 cells were treated with Icariside II (0, 25, 50 or 100 µM) for 9 h. EMSA was performed to determine the STAT3/DNA binding activity. N.S.; nonspecific binding. (E) U937 cells were treated with Icariside II (50 µM) for 0, 3, 6, 9 or 12 h. Western blotting was performed for phospho-STAT5 and STAT5. Representative results of three independent experiments are shown for each experiment.

We recently reported that Icariside II had the inhibitory effect on STAT3 activation in multiple myeloma cells [5]. To test the effect of Icariside II on U937 cells, immunoblotting analysis was performed (Fig. 4A and B). The addition of Icariside II reduced the phosphorylation of STAT3 in a dose- and time-dependent manner in U937 cells. Inhibitory effect of Icariside II on STAT3 activation was also found in HL-60 cells (Fig. 4C). Consistently, EMSA assay revealed that this herb compound significantly inhibited the STAT3/DNA binding activity in a dose-dependent manner (Fig. 4D). In contrast, Icariside II did not show significant effect on the phosphorylation of STAT5 (Fig. 4E).

### Icariside II inhibited phosphorylation of JAK2 and Src in U937 cells

**Figure 5:**
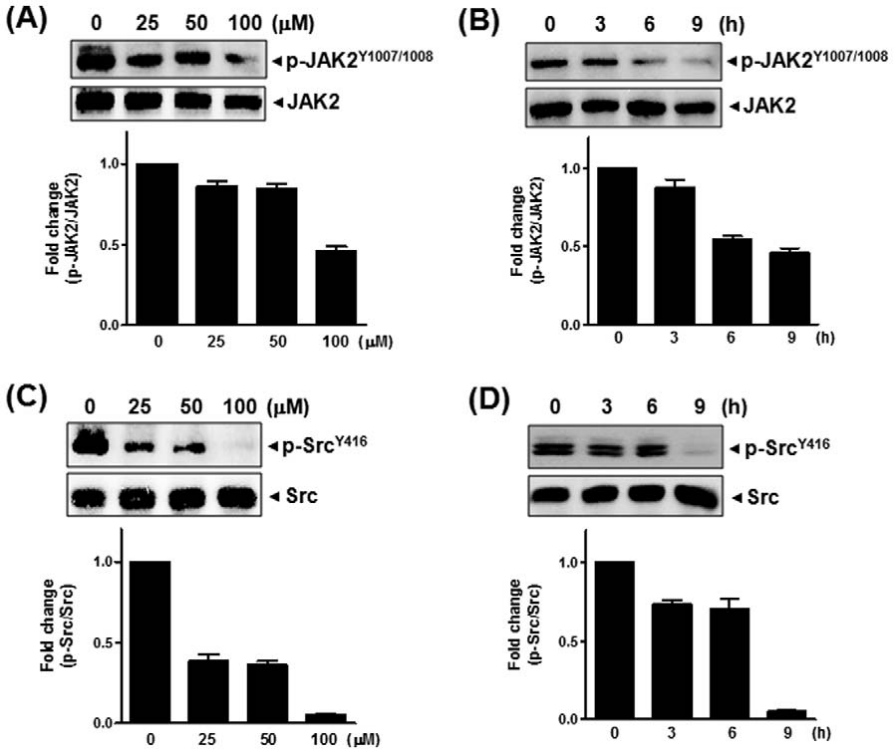
Effect of Icariside II on JAK2 and Src in U937 cells. (A and C) Cells were treated with Icariside II (0, 25, 50 or 100 µM) for 9 h. (B and D) Cells were treated with Icariside II (50 µM) for 0, 3, 6 or 9 h. Cell lysates were prepared and subjected to Western blotting for phospho-JAK2 and JAK2 (A and B), and phospho-Src and Src (C and D). Representative results of three independent experiments are shown for each experiment.

STAT3 is activated by cooperating with JAKs and/or directly by Src kinase [13]. Here, we again demonstrated that the treatment with Icariside II decreased the level of phopsho-JAK2, but not phospho-JAK1 (data not shown), in a dose- and time-dependent manner (Figs. 5A and B). Furthermore, the addition of Icariside II blocked the phosphorylation of Src in a dose- and time-dependent manner (Figs. 5C and D), indicating that Icariside II inactivates STAT3 signaling pathway through inhibiting JAK2 and Src in U937 cells.

### Protein tyrosine phosphatase (PTP) was involved in Icariside II-induced apoptosis in U937 cells

**Figure 6:**
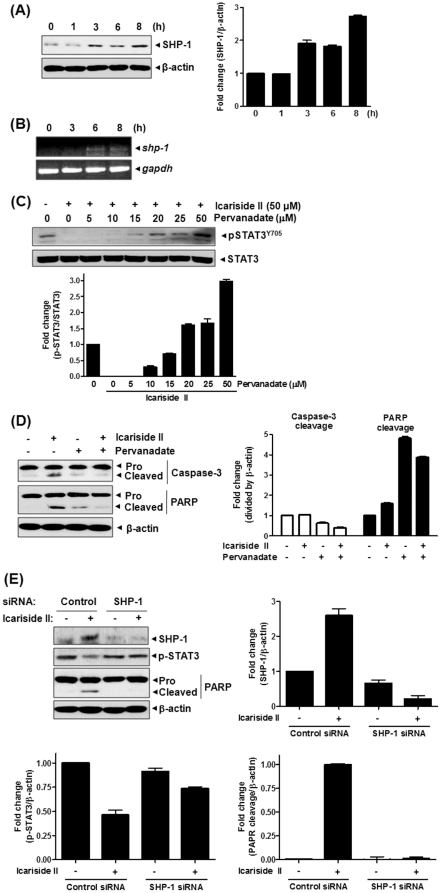
Involvement of protein tyrosine phosphatase SHP-1 in Icariside II-induced apoptosis in U937 cells. (A) Cells were treated with Icariside II (50 µM) for 0, 1, 3, 6 or 8 h. Cell lysates were prepared and subjected to Western blotting for SHP-1. (B) Total RNA from cells treated with Icariside II (50 µM) 0, 3, 6 or 8 h was extracted, and the mRNA level of SHP-1 was analyzed by RT-PCR. GAPDH was used as an internal control. (C) Cells were treated with Icariside II (50 µM) in the absence or presence of pervanadate (0, 5, 10, 15, 20, 25 or 50 µM) for 9 h. Cell lysates were prepared and subjected to Western blotting for phospho-STAT3 and STAT3. (D) Cells were treated with Icariside II (50 µM) and/or pervanadate (20 µM) for 9 h. Cell lysates were prepared and subjected to Western blotting for caspase-3 and PARP. Representative results of three independent experiments are shown for each experiment. (E) Cells were transiently transfected with either SHP-1 or scrambled siRNA (40 nM) for 48 h and then treated with Icariside II (50 µM) EP for 9 h. Western blotting was performed for SHP-1, phospho-STAT3 and PARP.

PTPs are known to induce dephosphorylation of protein tyrosine kinases (PTKs) including STAT family proteins [14] and the transient character of the tyrosine phosphorylation of JAK2 and STAT3 suggests the involvement of protein tyrosine phosphatases (PTPs) as negative regulators of this signaling pathway [15]. To test whether protein tyrosine phosphatase (PTP) was involved in Icariside II-induced apoptosis in U937 cells, immunoblotting analysis was performed. The treatment with Icariside II increased the expression of SHP-1, an upstream PTP of JAK2, in a time-dependent manner (Fig. 6A). Consistently, Icariside II also enhanced mRNA level of SHP-1 in a time-dependent manner in U937 cells (Fig. 6B). Conversely, PTP inhibitor sodium pervanadate overturned the Icariside II-mediated inactivation of STAT3 in a dose-dependent manner (Fig. 6C). Interestingly, pervanadate treatment also reversed the cleavages of caspase-3 and PARP induced by Icariside II (Fig. 6D). Furthermore, silencing SHP-1 using its specific siRNA significantly blocked STAT3 inactivation and apoptosis induced by Icariside II (Fig. 6E), suggesting that Icariside II induced apoptosis via inhibition of STAT3 in U937 cells.

### Icariside II induced apoptosis but did not affect phospho-JAK2 and SHP-1 in STAT3 inactive MM.1S cells

**Figure 7:**
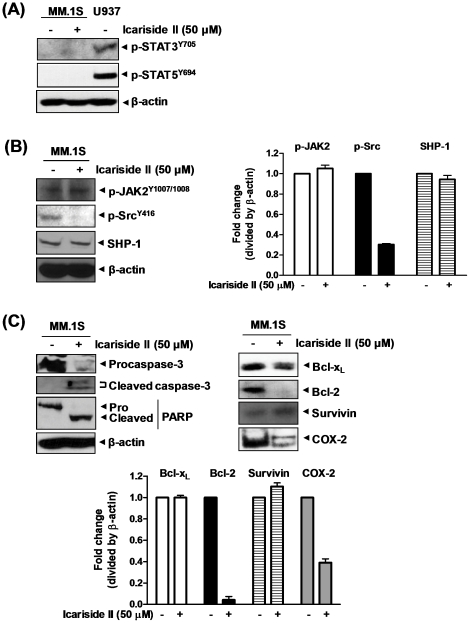
Effects of Icariside II on STAT3-related signaling in STAT3 inactive MM.1S cells. (A) MM.1S cells were treated with or without 50 µM Icariside II for 9 h. Cell lysates were prepared and subjected to Western blotting for phospho-STAT3 and phospho-STAT5. U937 cells were used as a control of phospho-STAT3 and 5. (B) MM.1S cells were treated with or without 50 µM Icariside II for 9 h. Cell lysates were prepared and subjected to Western blotting for phospho-JAK2, phospho-Src and SHP-1. (C) MM.1S cells were treated with or without 50 µM Icariside II for 24 h. Cell lysates were prepared and subjected to Western blotting for procaspase-3, PARP, bcl-x_L_, bcl-2, survivin and COX-2. Representative results of three independent experiments are shown for each experiment.

To further examine whether Icariside II specifically inhibits STAT3 signaling, we used STAT3 inactive MM.1S cells. The cells did not show any phosphorylation of STAT3 as well as STAT5 (Fig. 7A). Differently from STAT3 positive U937 cells, Icariside II treatment had no significant effect on phospho-JAK2 and SHP-1 expression in MM.1S cells (Fig. 7B), while Icariside II induced apoptosis by targeting bcl-2 and COX-2, but not bcl-x_L_ and survivin (Fig. 7C).

## Discussion

In the current study, the underlying mechanisms of Icariside II-induced anti-cancer activity were investigated in U937 AML cells. Apoptosis is the process of programmed cell death characterized by a series of morphological alterations including plasma and nuclear membrane blebbing and cell shringkage [16], and the molecular regulation such as caspase activation [17]. Here, we demonstrated that Icariside II significantly suppressed the viability of U937 cells, increased sub-G1 and TUNEL-positive cells and attenuated the expression of caspase-3, along with enhanced cleavage of PARP, indicating that Icariside II may exert its antitumor activity via enabling to induce apoptosis in U937 cells. Bcl-2 and inhibitor of apoptosis protein (IAP) family proteins are known as anti-apoptotic proteins [16], [18]. We also showed that Icariside II attenuated the expression levels of bcl-2, bcl-x_L_ and survivin in a time-dependent manner, further suggesting the Icariside II-induced apoptosis in U937 cells.

STAT3, one of member of the STAT family, is a transcriptional factor that can be activated by cytokines or growth factors in normal cellular responses. Recently, STAT3 has been considered as an important molecular target for cancer therapy due to its strong activation in various cancer cells including AML. Zhao and colleagues reported that sorafenib, a multikinase inhibitor, induced apoptosis in HL-60 AML cells by inhibiting Src kinase-mediated STAT3 phosphorylation [19]. Redell and colleagues reported that a novel small molecule STAT3 inhibitor, C188-9, suppressed G-CSF-induced STAT3 phosphorylation and apoptosis induction in AML cells, implying that STAT3 inhibition can be a valuable strategy for targeted therapies for AML [20].

In this study, we found that Icariside II suppressed the phosphorylation of constitutively active STAT3 in AML cell lines U937 and HL-60. In contrast, Icariside II did not alter the phosphorylation of STAT5 in U937 cells, indicating the specificity of Icariside II for STAT3 in AML cells. Furthermore, Icariside II treatment dramatically inhibited the transcriptional activity of STAT3 in gel shift assay by disturbing the binding of STAT3/DNA in U937 cells, suggesting that constitutively active STAT3 activity was not promoted by Icarisde II to undergo a dephosphorylation event. Moreover, a significant reduction of phospho-JAK2 and Src, the upstream tyrosine kinases of STAT3, was observed in Icariside II-treated U937 cells in a dose- and time-dependent manner, which was similarly supported by our previous report in multiple myeloma cells [5]. Thus, the anti-cancer activities of Icariside II can be exerted by its pleiotropic effects on the multiple targets including STAT3 as well as JAK2, Src, and anti-apoptotic bcl-2, bcl-x_L_, survivin and COX-2 in U937 cells.

Activation of tyrosine kinases including STAT3 is regulated by balancing with PTPs. Various PTPs have been reported to be responsible for dephosphorylation of STAT3, including SHP-1 [21], SHP-2 [22], T cell (TC)-PTP [23], phosphatase and tensin homolog (PTEN) [24], suppressor of cytokine signaling (SOCS)-1 [25]. In our study, Icariside II treatment increased SHP-1 expression at protein and mRNA levels in U937 cells. Importantly, the co-treatment with pervanadate (a general PTP inhibitor) effectively blocked Icariside II-induced STAT3 inactivation as well as apoptosis, strongly indicating that Icariside II, via affecting SHP-1, had a negative effect on STAT3 for the induction of apoptosis in U937 cells. Similarly, STAT3 targeted efficacies were reported by potent natural compounds such as ursolic acid [26], guggulsterone [27], genipin [28] and compound K [29] in various cancer cells.

Of interest, Icariside II also induced apoptosis in STAT3 inactive MM.1S multiple myeloma cells. Nonetheless, Icariside II treatment did not target bcl-x_L_ and survivin, and phosphorylation of JAK2 compared to STAT3 active U937 cells. Furthermore, highly expressed SHP-1 protein was not changed by Icariside II treatment in MM.1S cells. These results suggest Icariside II induced apoptosis through an alternative pathway in STAT3 inactive MM.1S cells.

In conclusion, Icariside II suppressed the growth of U937 cells, by sensitizing the cells to apoptosis, manifested by increasing TUNEL-positive cells and accumulation of sub-G1 population in U937 AML cells. Also, Icariside II treatment increased the expression caspase-3, cleavage of PARP, and decreased bcl-x_L_ and survivin. In addition, suppressed the activation of STAT3 and JAK2 and enhanced SHP-1 expression in U937 cells. Moreover, blocking SHP-1 by sodium pervanate attenuated the Icariside II-induced STAT3 inhibition and PARP cleavage in U937 cells. These findings suggested that Icariside II can induce apoptosis via inactivation of STAT3-related signaling pathway in AML U937 cells.

## Materials and Methods

### Isolation of Icariside II

Icariside II (Fig. 1A) was isolated from *Epimedium koreanum* as previously described previously [6].

### Cell lines

U937, HL-60 and MM.1S cells were purchased from American Type Culture Collection (ATCC) (Rockville, MD) and maintained in RPMI 1640 containing antibiotic and antimycotic solution with 10% fetal bovine serum.

### Cell proliferation assay

The anti-proliferative effect of Icariside II was determined by 3-(4,5-dimethylthiazol-2yl)-2,5-diphenyltetrazolium bromide (MTT) assays. Cells (5×10^3^ cells/well) were seeded in a 96-well plate, and treated with Icariside II (0, 25 or 50 µM) for 0, 24, 48 or 72 h. The treated cells were incubated with medium containing 5 mg/ml of MTT for 2 h at 37°C and then solubilized by 200 µl of lysis solution. The absorbance was read on a microplate reader (Molecular Devices E-max) at 570 nm.

### Cell cycle analysis

Cells were exposed to Icariside II (50 µM) for 0, 1 or 24 h and fixed in 70% cold ethanol overnight at −20°C. The fixed cells were centrifuged, washed, resuspended in 100 µl of PBS containing 10 µl of RNase A (10 mg/ml) and incubated for 1 h at 37°C, and stained by adding 900 µl of propidium iodie (PI) (50 µg/ml) for 30 min at room temperature in dark. The DNA contents of stained cells were analyzed using Cellquest Software with a FACSCalibur flow cytometry (BD Biosciences, San Jose, CA).

### Terminal deoxynucleotidyl transferase dUTP nick end labeling (TUNEL) assay

Individual apoptotic cell death was observed using DeadEnd™ fluorometric TUNEL assay kit (Promega, Madison, WI) as described by manufacturer. Cells were treated with 50 µM Icariside II for 0, 12 or 24 h and washed with cold PBS. The cells were fixed with 4% paraformaldehyde for 30 min, washed twice with PBS for 2 min, resuspended in permeabilization solution (0.1% Triton X-100 and 0.1% Sodium citrate) for 4°C overnight, and incubated with 25 µl of TUNEL assay mixture (Sigma, St. Louis, MO) for 60 min at 37°C in a humidified atmosphere in dark. After washing 3 times in PBS for 2 min and filtering, the cells were analyzed by the flow cytometry.

### Western blotting

Whole-cell extracts were lysed in lysis buffer [20 mM tris (pH 7.4), 250 mM NaCl, 2 mM EDTA (pH 8.0), 0.1% Triton X-100, 0.01 mg/ml aprotinin, 0.003 mg/ml leupeptin, 0.4 mM phenylmethylsulfonyl fluoride (PMSF), and 4 mM NaVO_4_]. Lysates were then spun at 13,000× g for 15 min to remove insoluble material and resolved on a 10% SDS gel. After electrophoresis, the proteins were electrotransferred to a nitrocellulose membrane, blocked with 5% nonfat milk, and probed with antibodies against caspase-3, PARP, bcl-2, bcl-x_L_, survivin, COX-2, SHP-1 (Santa Cruz Biotechnologies, Santa Cruz, CA), phospho-STAT3, STAT3, phospho-STAT5, STAT5, phospho-JAK2, JAK2, phospho-Src and Src (Cell Signaling, Danvers, MA) overnight. The blots were washed, exposed to horseradish peroxidase (HRP)-conjugated secondary antibodies for 2 h, and finally examined by enhanced chemiluminescence (ECL) (GE Health Care Bio-Sciences, Piscataway, NJ). Band intensities were quantified using NIH Image-J software.

### Electrophoretic mobility shift assay (EMSA)

The STAT3/DNA binding was analyzed by electrophoretic mobility shift assay (EMSA) using Gelshift Chemiluminescent EMSA kit (Active Motif, Carlsbad, CA). Nuclear extracts were prepared and incubated with STAT3 consensus oligonucleotides (5′-GAT CCT TCT GGG AAT TCC TAG ATC-3′) (Santa Cruz Biotechnologies, Santa Cruz, CA). The DNA/protein complex formed was separated from free oligonucleotides on 5% native polyacrylamide gels. Chemiluminescent detection was performed using ECL reagents according to the vendor's protocols (GE Health Care Bio-Sciences, Piscataway, NJ).

### Reverse transcription-PCR (RT-PCR)

Total RNA was extracted by using Trizol reagent (Invitrogen, Carlsbad, CA) according to the manufacturer's instructions. cDNA was synthesized from 1 µg of total RNA and subjected to PCR reaction by using Superscript One Step reverse transcription-PCR (RT-PCR) kit (Invitrogen, Carlsbad, CA). The PCR conditions were 30 cycles of 94°C for 15 s, 55°C for 30 s, and 72°C for 1 min. The primer sequences were as follows: *shp-1* (forward primer 5′-AAT GCG TCC CAT ACT GGC CCG A-3′; reverse primer 5′-CCC GCA GTT GGT CAC AGA GT-3′) and *gapdh* (forward primer 5′-TCA CCA TCT TCC AGG AGC GA-3′; reverse primer 5′-CAC AAT GCC GAA GTG GTG GT-3′). PCR products were run on 2% agarose gel and then stained with ethidium bromide. Stained bands were visualized under UV light and photographed.

### siRNA transfection

U937 cells were transiently transfected with STAT3-siRNA or control-siRNA (40 nM) (Santa Cruz Biotechnology, Santa Cruz, CA) for 48 h using INTERFERin™ transfection reagent (Polyplus-transfection Inc., New York, NY) according to manufacturer's protocols.

### Statistical analysis

All data were expressed as means ± standard deviation (S.D.) of three independent experiments.
